# Impact of Home Health Care on End-of-Life Outcomes for Medicare Beneficiaries Across Racial and Ethnic Groups

**DOI:** 10.1093/geroni/igaf122.1323

**Published:** 2025-12-31

**Authors:** Olga Jarrín, Babatope Ogunjesa, Andre Rosario, Dione Sandiford, Roland Thorpe, Anum Zafar, Haiqun Lin, Paul Duberstein

**Affiliations:** Rutgers, The State University of New Jersey, New Brunswick, New Jersey, United States; University of Illinois Urbana-Champaign, Champaign, Illinois, United States; Rutgers University, New Brunswick, New Jersey, United States; Rutgers University, Newark, New Jersey, United States; Johns Hopkins Bloomberg School of Public Health, Baltimore, Maryland, United States; Rutgers, The State University of New Jersey, New Brunswick, New Jersey, United States; Rutgers, The State University of New Jersey, Newark, New Jersey, United States; Rutgers University, New Brunswick, New Jersey, United States

## Abstract

End-of-life care is crucial for Medicare beneficiaries, yet racial and ethnic disparities persist in access and use of hospice care and place of death. The aim of this study was to evaluate the impact of home health care on racial/ethnic inequities in end-of-life outcomes for Medicare beneficiaries with and without dementia. Analyzing patterns in 2,065,300 beneficiaries who died in 2019, logistic models were used to explore the impact of home health care use during the last three years of life on end-of-life care outcomes. Home health care use was associated with a lower predicted probability of hospital death without hospice and a higher probability of home death with hospice across all racial/ethnic groups. Home health care use (vs. no home health care use) increased the probability of home death with hospice more for White, Asian American/Pacific Islander, and American Indian and Alaska Native beneficiaries compared to Hispanic and Black beneficiaries. Among patients with dementia, similar trends were observed, but the effect was smaller relative to patients without dementia. For example, among Black decedents with dementia, home health care was associated with a 5.1% increase in the probability of home death with hospice vs. an 8.2% increase among those without dementia. This study underscores the role of home health care in enhancing end-of-life quality, especially in facilitating the use of home hospice care. However, targeted interventions to ensure equitable access to hospice care are needed to optimize end-of-life care and meet the diverse needs of Medicare beneficiaries.

